# Optimized workflow with hybrid (very) high-power short-duration radiofrequency ablation renders point-by-point pulmonary vein isolation as fast and effective as cryoballoon ablation

**DOI:** 10.1007/s10840-025-01982-4

**Published:** 2025-01-11

**Authors:** Marco Fusaroli, Mark G. Hoogendijk, Rohit E. Bhagwandien, Sip A. Wijchers, Nick van Boven, Bakthawar K. Mahmoodi, Sing-Chien Yap

**Affiliations:** 1 Department of Cardiology, Thorax Center, Cardiovascular Institute, Erasmus MC, Rotterdam, the Netherlands; 2 Department of Experimental and Clinical Medicine, School of Human Health Sciences, Careggi University Hospital, University of Florence, Florence, Italy; 3 Department of Cardiology, Franciscus Gasthuis & Vlietland, Rotterdam, the Netherlands

**Keywords:** Atrial fibrillation, Cryoballoon ablation, High power short duration, Very high power short duration, Atrial arrhythmia recurrence

## Abstract

**Introduction:**

A hybrid approach with very high-power short-duration (vHPSD) posteriorly and ablation-index guided HPSD (50 W) anteriorly seems to be an optimal balance between efficiency and effectiveness for point-by-point pulmonary vein isolation (PVI). The aim of the current study is to compare vHPSD/HPSD ablation to cryoballoon ablation (CBA) in patients with symptomatic atrial fibrillation (AF).

**Methods and results:**

In this retrospective single-center study, we identified 110 consecutive patients who underwent their first PVI with either vHPSD/HPSD (*n* = 54) or CBA (*n* = 56). We compared procedural efficacy, efficiency, safety, and long-term outcomes. Baseline characteristics of both groups were comparable; however, patients in the vHPSD/HPSD group had larger left atrial volume index (35, IQR 27–45 vs. 28, IQR 21–36 ml/m^2^, *P* = 0.005). Complete PVI was achieved in all patients except two CBA cases (100% vs. 96.4%, *P* = 0.50). First-pass isolation rate was 79.6% in the hybrid group. Procedure times were similar between groups (53, IQR 47–63 vs. 55, IQR 49–65 min, *P* = 0.35), but fluoroscopy time was shorter in the vHPSD/HPSD group (3.9 [2.7, 5.6] vs. 11.9 [9.3, 14.9] min, *P* < 0.001). There were 3 temporary phrenic nerve palsies (5.4%) in the CBA group which resolved within 1 year. The 1-year freedom from any atrial tachyarrhythmias after a single procedure was similar between groups (68.5% vs. 73.2%, *P* = 0.56). During repeat procedure, the durability of PVI was comparable.

**Conclusions:**

The use of vHPSD/HPSD ablation renders point-by-point PVI as fast and effective as CBA. Furthermore, it has lower radiation exposure compared to CBA.

**Graphical abstract:**

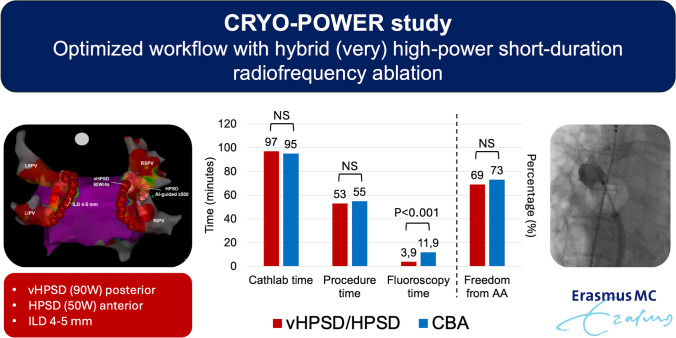

## Introduction

The indication for pulmonary vein isolation (PVI) has expanded from the treatment of drug-refractory paroxysmal or persistent atrial fibrillation (AF) to a first-line option in patients with paroxysmal AF [1]. It is expected that this will result in more patients with AF being treated with catheter ablation. To cope with this increase, procedural efficiency is becoming more relevant to address waiting lists. Currently, there are many techniques available to achieve PVI, including cryoballoon ablation (CBA), radiofrequency (RF) catheter ablation (RFCA), and pulsed field ablation (PFA). The ADVENT trial has demonstrated that the arrhythmia outcome of the different modalities were comparable [2]. PFA was associated with shorter procedures times than thermal ablation but required a longer duration of fluoroscopy. When comparing the 2 thermal ablation modalities, conventional RFCA is associated with lower fluoroscopy times but longer procedures times and higher risk of repeat ablations in comparison to CBA [3, 4]. Within RFCA, there has been a shift towards the use of higher power settings (45–70 W) over shorter duration, i.e., high-power short-duration (HPSD), which has resulted in better procedural effectiveness and shorter procedure durations with comparable safety [5–9]. The QDOT Micro ablation catheter (Biosense Webster Inc, Irvine, CA) allows temperature- and flow-controlled ablation and very high-power short-duration (vHPSD) ablation (90 W/4 s) [10–13]. Although the use of vHPSD for PVI is fast, there seems to be lower rates of first-pass isolation probably due to the more shallow ablation lesion characteristics [13]. Some authors suggested to use a hybrid approach of vHPSD (90 W/4 s) posteriorly and ablation index (AI)-guided HPSD (50W) anteriorly [13–15]. We optimized our workflow for point-by-point PVI using this hybrid approach to improve procedural efficiency but to retain a high first-pass isolation rate. First-pass isolation reflects an accurate, effective, and contiguous point-by-point lesion set applied in the first encirclement. The aim of the present study is to compare the procedural efficiency, safety and arrhythmia outcome of this hybrid vHPSD/HPSD approach for point-by-point PVI to a single-shot technique of CBA.

## Methods

### Study population

This was a retrospective, single-center, non-randomized study including consecutive adult patients with symptomatic AF who underwent their first PVI (without additional lesions) with hybrid vHPSD/HPSD RFCA ablation or CBA between August 2022 and July 2023 in the Erasmus MC. The choice of the technique was mainly based on the operator preference, pulmonary vein (PV) anatomy (presence of common ostium), and logistics (availability of general anesthesia). Patients who participated in a clinical study requiring additional study-related mapping were excluded. No additional lesions were delivered, except from PVI. The study was approved by the Institutional Review Board (CRYO-POWER, METC-2024–0459), and this study was not subjected to the Dutch Medical Research Involving Human Subjects Act. The study was carried out according to the ethical principles for medical research involving human subjects established by Declaration of Helsinki (2013), protecting the privacy of all the participants and the confidentiality of their personal information.

### Periprocedural management

All patients were administered oral anticoagulation for a minimum of 4 weeks before undergoing ablation. Direct-acting oral anticoagulants were continued during the procedure. Patients using vitamin K antagonists had a target INR between 2.0 and 2.5 on the day of the procedure. To exclude left atrial thrombi, all patients underwent transesophageal echocardiogram just before the procedure.

### Cryoballoon ablation protocol

The CBA procedures were usually performed under deep sedation. CBA procedures were performed by two experienced CBA operators. Two short introducer sheaths (8F, 6F) were placed in the right common femoral vein using ultrasound guidance. An intravenous heparin bolus of 5000 IE was given after vascular access. A decapolar catheter was placed in the coronary sinus. A single transseptal puncture with a SL1 sheath (Swartz, Abbott, Abbott Park, IL) was performed under the guidance of transesophageal echocardiography. The SL1 sheath was replaced by a large steerable sheath (either FlexCath Advance, Medtronic, Minneapolis, MN; or POLARSHEATH, Boston Scientific, Marlborough, MA). A second intravenous heparin bolus was given to achieve a target activated clotting time of ≥ 300 s. Then, a CBA catheter (Arctic Front Advance Pro, Medtronic; POLARx, Boston Scientific; POLARx FIT, Boston Scientific) was inserted in the left atrium (LA). PV potentials were recorded using a 20-mm circular inner lumen mapping catheter with 8 electrodes (Achieve, Medtronic; POLARMAP, Boston Scientific). After optimal PV occlusion was achieved, assessed by contrast injection, cryoablation was started. A time-to-isolation (TTI)-guided ablation protocol was used. The freeze duration was 180 s if TTI was < 60 s; otherwise, a 240-s freeze cycle was employed. No bonus freeze was employed routinely. PVI was confirmed by entrance and exit block at the end of the procedure. During cryoablation of the right-sided PVs, high-output right phrenic nerve stimulation was performed. Diaphragmatic excursion was assessed during cryoablation. Whenever the diaphragmatic excursions decreased cryoablation was immediately terminated. No waiting time was employed. Vascular access was closed using a purse-string suture.

### Radiofrequency ablation protocol

**Fig. 1:**
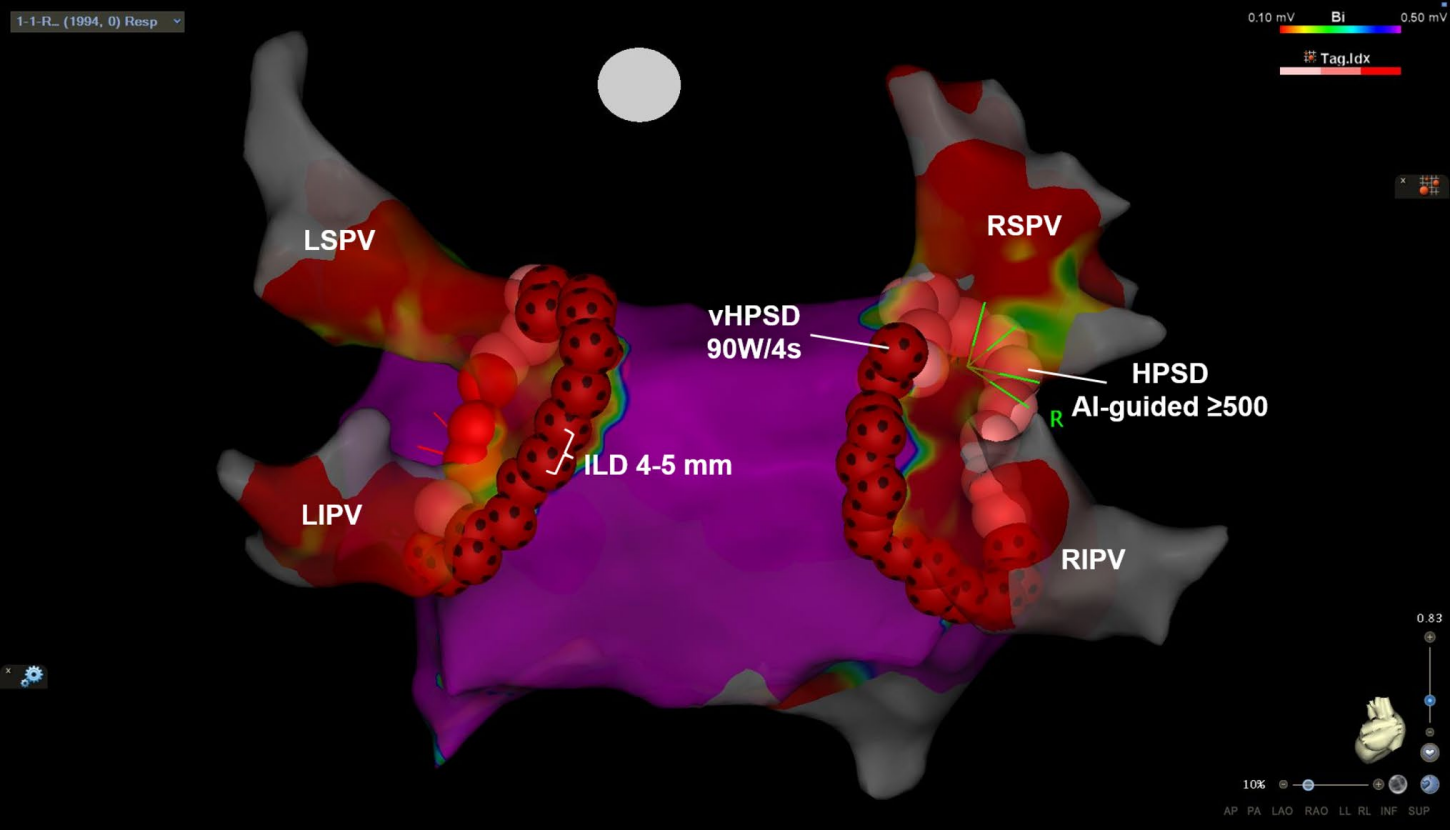
Representative example of WACA with a hybrid vHPSD/HPSD approach (posterior view). QMODE + setting (90 W/4 s) was used on the posterior wall, AI-guided HPSD (50 W) was used anteriorly. The inter-lesion distance was 4–5 mm

All RFCA procedures were performed under general anesthesia using low tidal volumes (3–3.5 ml/kg). RFCA procedures were performed by two experienced operators. Two short introducer sheaths (2 × 8F) were placed in the right common femoral vein using ultrasound guidance. A heparin bolus of 5000 IE was given after vascular access. A transseptal puncture with a SL1 sheath (Swartz, Abbott, Abbott Park, IL) and BRK1-needle was performed under the guidance of transesophageal echocardiography. A multipolar mapping catheter (PENTARAY, Biosense Webster) was placed in the left superior PV through the SL1 sheath. A second intravenous heparin bolus was given to achieve a target activated clotting time of ≥ 300 s. The septum was then recrossed with an Agilis NxT steerable sheath (Abbott) and the QDOT Micro catheter was placed in the LA. A 3-dimensional electro-anatomical map of the LA was created with the PENTARAY catheter using the CARTO3 system (Biosense Webster). After creation of the LA map, ipsilateral point-by-point wide area circumferential ablation (WACA) was performed using an inter-lesion distance (ILD) of 4 to 5 mm (Fig. 1). We used QMODE + setting (90 W, 4 s) posteriorly and AI-guided QMODE setting (50 W, target ablation index 500) anteriorly with the nGEN RF generator (Biosense Webster). The target contact force range during ablation was between 5 to 15 g. Ablation was performed during atrial pacing with the PENTARAY catheter at a cycle length of 600 ms (from right sided PVs during left-sided WACA, from LAA during right-sided WACA). After completion of the WACA, PV isolation was confirmed with the PENTARAY catheter. If WACA failed to achieve PVI, additional RFCA applications focused on earliest activation in the PV antrum (usually activation breakthrough of epicardial connections) were performed to achieve PVI. For additional lesions we usually chose the QMODE setting (50 W) to achieve deeper ablation lesions. No waiting time was employed. Vascular access was closed using a purse-string suture.

### Follow-up

Patients had their outpatient clinical visit at 3, 6, and 12 months after their index ablation. They received 24-h Holter monitoring at these visits. Antiarrhythmic drugs were continued until the first outpatient visit at 3 months. If there was no atrial arrhythmia recurrence, antiarrhythmic drugs were discontinued as much as possible in agreement with the patient. Continuation of oral anticoagulation was based on the individual CHA_2_DS_2_-VASc score.

### Endpoints

The primary endpoint was the procedural efficiency as measured by achieving complete PVI and procedural characteristics (i.e., procedure time, cathlab time, fluoroscopy time). The procedure time was defined as the skin-to-skin time. The cathlab time was defined as the door-to-door time. Secondary endpoints were procedural safety and the 1-year clinical outcome. Clinical outcome was defined as the 1-year freedom of atrial tachyarrhythmias and the freedom of AF. Atrial tachyarrhythmias (> 30 s) had to be documented on an ECG or Holter monitor. We used a blanking period of 8 weeks post-ablation in agreement with the recent international expert consensus document [16]. The safety profile was assessed in terms of both periprocedural complications (within 7 days of the procedure), including phrenic nerve palsy, stroke, transient ischemic attack, vascular complications, cardiac tamponade, and death, as well as long-term complications over a 12-month period, such as persistent phrenic nerve palsy (PNP), pulmonary vein stenosis, and atrial esophageal fistula.

### Statistical analysis

Continuous variables are expressed as mean ± standard deviation or as median (25th; 75th percentile) and were compared using the Student’s *t* test or the Mann–Whitney *U* test, as appropriate. Categorical variables were presented as percentages and compared using the chi-square test or Fisher’s exact test, as appropriate. Survival analysis for arrhythmia-free outcomes was performed using the Kaplan–Meier method, with differences between groups evaluated by the log-rank test. Statistical significance was set at a *P* value of less than 0.05. All analyses were performed using SPSS software, version 28.0.1.0 (SPSS Inc., Chicago, IL, USA).

## Results

### Baseline characteristics

A total of 110 patients underwent their first PVI for symptomatic AF with either hybrid vHPSD/HPSD (*n* = 54) or CBA (*n* = 56) in the study period. The baseline characteristics of both groups were comparable, although patients in the hybrid group had larger LA dimensions (LA volume index, 35 [27, 45] vs. 28 [21, 36.3] ml/m^2^, *P* = 0.005) (Table 1).

**Table 1:** Baseline patient characteristics

Variable	Hybrid vHPSD/HPSD (*n* = 54)	CBA (*n* = 56)	*P* value
Age (years)	61 (57, 72)	61 (56, 69.8)	0.70
Male sex	35 (64.8%)	34 (60.7%)	0.66
BMI (kg/m^2^)	28.5 ± 3.9	27 ± 4.3	0.06
Obesity (BMI ≥ 30 kg/m^2^)	20 (37%)	16 (28.6%)	0.34
Dyslipidemia	18 (33.3%)	16 (28.6%)	0.59
CHF history	6 (11.1%)	9 (16.1%)	0.45
Hypertension	26 (48.1%)	26 (46.4%)	0.86
Diabetes	4 (7.4%)	6 (10.7%)	0.74
Stroke/TIA history	4 (7.4%)	2 (3.6%)	0.43
Vascular disease history - CAD - PAD	8 (14.8%) 8 (14.8%) 1 (1.9%)	11 (19.6%) 9 (16.1%) 2 (3.6%)	0.50 0.86 1.00
CHA₂DS₂-VASc score - 0 - 1 - 2 - 3 - ≥ 4	2 (1, 3) 11 (20.4%) 14 (25.9%) 14 (25.9%) 9 (16.7%) 6 (11.1%)	2 (1, 3) 12 (21.4%) 14 (25%) 15 (26.8%) 5 (8.9%) 10 (17.9%)	0.96 0.89 0.91 0.92 0.22 0.32
Valvular heart disease (≥ mod.)	4 (7.4%)	6 (10.7%)	0.74
Nonischemic cardiomyopathy	3 (5.6%)	0 (0%)	0.12
Hyperthyroidism	2 (3.7%)	1 (1.8%)	0.62
COPD	4 (7.4%)	3 (5.4%)	0.71
OSAS	0 (0%)	3 (5.4%)	0.24
CKD-EPI eGFR (ml/min) - Normal function (≥ 90 ml/min) - Mild dysfunction (89–60 ml/min) - ≥ Moderate dysfunction (< 60 ml/min)	79.8 ± 17.5 18 (33.3%) 28 (51.9%) 8 (14.8%)	78.1 ± 21.4 19 (33.9%) 25 (44.6%) 12 (21.4%)	0.64 0.95 0.45 0.37
Type of AF			
- Paroxysmal - Persistent	41 (75.9%) 13 (24.1%)	43 (78.6%) 12 (21.4%)	0.74 0.74
EHRA classification			
- I - IIa - IIb - III - IV	0 (0%) 19 (35.2%) 25 (46.3%) 9 (16.7%) 1 (1.8%)	0 (0%) 14 (25.0%) 26 (46.4%) 15 (26.8%) 1 (1.8%)	- 0.24 0.99 0.20 1.00
LAVI (ml/m^2^)	35 (27, 45)	28 (21, 36)	0.005
Left ventricular function			
- Normal function (EF ≥ 50%) - Mild dysfunction (EF 40–49%) - Moderate dysfunction (EF 30–39%) - Severe dysfunction (EF < 30%)	48 (88.9%) 5 (9.3%) 1 (1.9%) 0 (0%)	51 (91.1%) 4 (7.1%) 1 (1.8%) 0 (0%)	0.70 0.74 1.00 -
CIED - Pacemaker - ICD	1 (1.9%) 0 (0%) 1 (1.9%)	3 (5.4%) 3 (5.4%) 0 (0%)	0.62 0.24 0.49
DOAC	51 (94.4%)	54 (96.4%)	0.68
VKA	3 (5.6%)	2 (3.6%)	0.68
Antiarrhythmic drugs - Class I - Class II - Class III - Sotalol - Amiodarone - Class IV - Digoxin	48 (88.9%) 13 (24.1%) 22 (40.7%) 22 (40.7%) 14 (25.9%) 8 (14.8%) 4 (7.4%) 3 (5.6%)	55 (98.2%) 11 (19.6%) 28 (50%) 30 (53.6%) 22 (39.3%) 8 (14.3%) 0 (0%) 3 (5.4%)	0.06 0.57 0.33 0.18 0.14 0.94 0.06 1.00

Values are presented as median (25th; 75th percentile), mean ± standard deviation or as count (%)

*AF* atrial fibrillation, *BMI* body mass index, *CAD* coronary artery disease, *CBA* cryoballoon ablation, *CHF* congestive heart failure, *CIED* cardiac implantable electronic device, *CKD-EPI* chronic kidney disease epidemiology collaboration, *COPD* chronic obstructive pulmonary disease, *DOAC* direct-acting oral anticoagulant, *EF* ejection fraction, *eGFR* estimated glomerular filtration rate, *EHRA* European Heart Rhythm Association, *HPSD* high-power short-duration, *ICD* implantable cardioverter defibrillator, *LA* left atrium, *LAVI* left atrial volume index, *OSAS* obstructive sleep apnea syndrome, *PAD* peripheral artery disease, *TIA* transient ischemic attack, *vHPSD* very high-power short-duration, *VKA* vitamin K antagonists

### Procedural characteristics and outcome

**Fig. 2:**
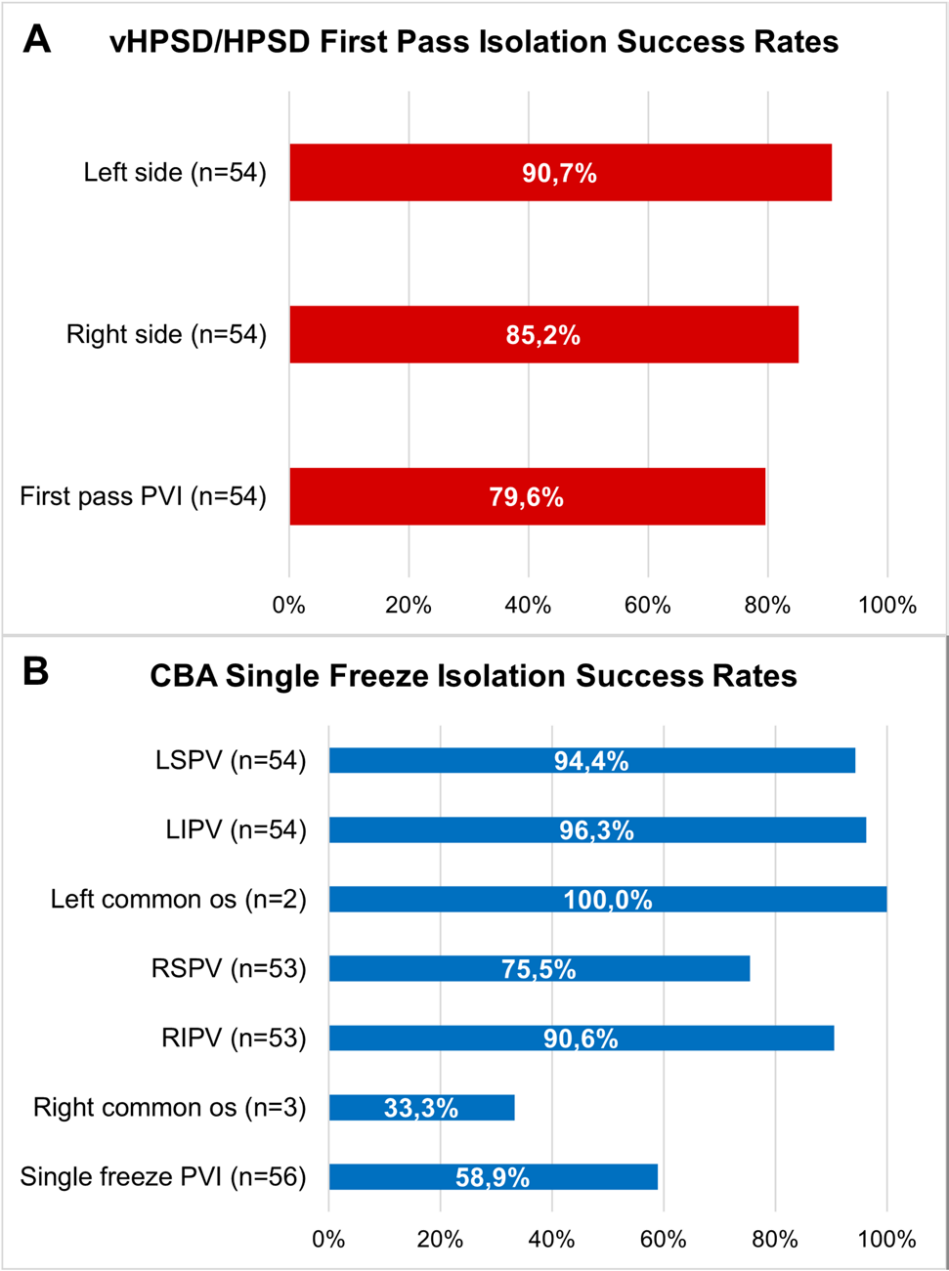
First-pass isolation in the hybrid vHPSD/HPSD group (**A**) and single freeze isolation rates in the CBA group (**B**)

The procedural characteristics for both groups are detailed in Table 2. All procedures in the hybrid vHPSD/HPSD group were performed under general anesthesia, while most CBA procedures (78.6%) were conducted under deep sedation. There were no significant differences in procedure time (53 [47, 63] vs. 55 [49, 65] min, *P* = 0.35) and total cathlab time (97 [88, 105] vs. 95 [84, 103] min, *P* = 0.51) between both groups. However, the hybrid vHPSD/HPSD group had shorter fluoroscopy times (3.9 [2.7, 5.6] vs. 11.9 [9.3, 14.9] min, *P* < 0.001) and lower radiation exposure (239.1 [131.1, 480.9] vs. 492.6 [267.8, 950.5] cGycm^2^, *P* < 0.001). There were more patients with a left common ostium in the hybrid vHPSD/HPSD group in comparison to the CBA group (24.1% vs. 3.6%, *P* = 0.002). While every patient in the hybrid vHPSD/HPSD group achieved successful isolation of all PVs, two patients in the CBA group did not experience complete PVI (100% vs. 96.4%, *P* = 0.50) due to the occurrence of acute PNP. This resulted in a not isolated right inferior PV and right common PV. Regarding periprocedural complications, no complications were observed in the hybrid vHPSD/HPSD group, whereas three patients in the CBA group experienced PNP (0% vs. 5.4%, *P* = 0.24). The first-pass isolation rate (79.6% patient-level) and single freeze rate (88.6% PV level; 58.9% patient level) were relatively high for the vHPSD and CBA cohort, respectively (Fig. 2).

**Table 2:** Procedural characteristics

Variables	Hybrid vHPSD/HPSD (*n* = 54)	CBA (*n* = 56)	*P* value
Presenting rhythm			
- Sinus rhythm - Atrial fibrillation	42 (77.8%) 12 (22.2%)	46 (82.1%) 10 (17.9%)	0.57 0.57
Type of anesthesia			
- Deep sedation - General anesthesia	0 (0%) 54 (100%)	44 (78.6%) 12 (21.4%)	< 0.001 < 0.001
Cathlab time (min)	97 (88, 105)	95 (84, 103)	0.51
Procedure time (min)	53 (47, 63)	55 (49, 65)	0.35
Fluoroscopy time (min)	3.9 (2.7, 5.6)	11.9 (9.3. 14.9)	< 0.001
Radiation dose (mGy)	17 (10, 41)	44 (22, 85)	< 0.001
DAP (cGycm^2^)	239.1 (131.2, 480.9)	492.6 (267.8, 950.5)	< 0.001
Total ablation time (s)	516 (429, 629)	811 (720, 960)	< 0.001
In hospital complications - Phrenic nerve palsy - Stroke - TIA - Vascular complication - Cardiac Tamponade - Death	0 (0%) 0 (0%) 0 (0%) 0 (0%) 0 (0%) 0 (0%) 0 (0%)	3 (5.4%) 3 (5.4%) 0 (0%) 0 (0%) 0 (0%) 0 (0%) 0 (0%)	0.24 0.24 - - - - -

Values are presented as median (25th, 75th percentile) or as count (%)

*CBA* cryoballoon ablation, *DAP* dose area product, *HPSD* high-power short-duration, *TIA* transient ischemic attack, *vHPSD* very high-power short-duration

### Long-term clinical outcome

**Fig. 3:**
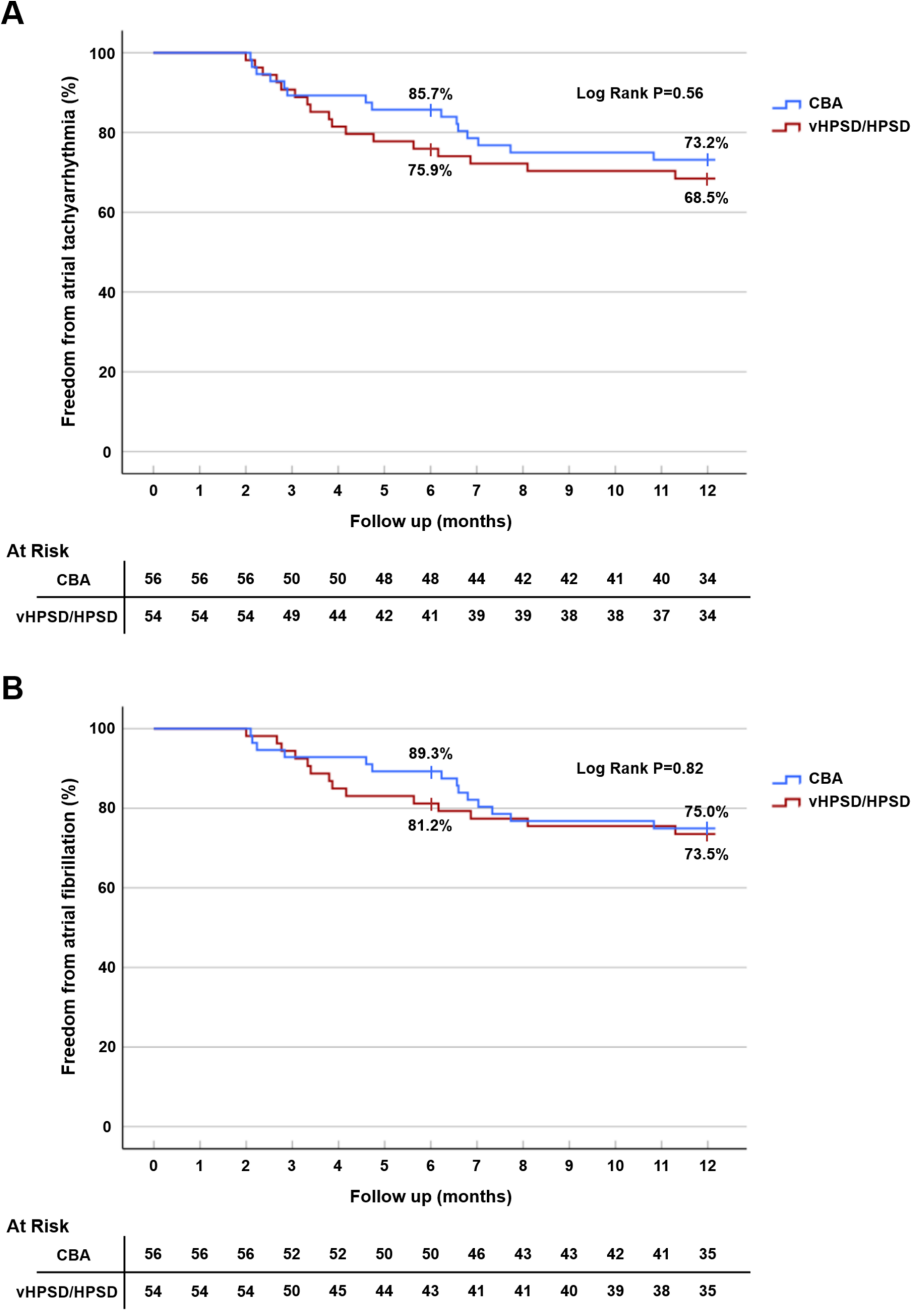
Freedom from atrial tachyarrhythmias (**A**) and atrial fibrillation (**B**) after a single procedure with a blanking period of 8 weeks

The mean duration of follow-up was 406 ± 48 days after the index procedure. The incidence of recurrence of atrial arrhythmia, after a blanking period of 2 months, was comparable between groups (Table 3). The 1-year freedom from any atrial tachyarrhythmias and AF was 68.5% (vHPSD/HPSD) vs. 73.2% (CBA), log-rank *P* = 0.56; and 73.5% (vHPSD/HPSD) vs. 75% (CBA), log-rank *P* = 0.82, respectively (Fig. 3). There was also no difference between the use of antiarrhythmic drugs at 1 year (Table 3), although sotalol was more often used in the CBA group (17.9% vs. 5.6%, *P* = 0.046). In total, 15 patients underwent a repeat ablation within the first year after the index procedure (vHPSD/HPSD, *n* = 6; CBA, *n* = 9).

**Table 3:** Long-term clinical outcome

Variables	Hybrid vHPSD/HPSD (*n* = 54)	CBA (*n* = 56)	*P* value
Duration of follow-up (days)	406 ± 49	406 ± 46	0.98
AT recurrence - AF - AFL - SVT	17 (31.5%) 14 (25.9%) 1 (1.9%) 3 (5.6%)	15 (26.8%) 14 (25%) 4 (7.1%) 1 (1.8%)	0.59 0.91 0.36 0.36
Electrical cardioversion	7 (13.0%)	5 (8.9%)	0.50
Repeated ablation	6 (11.1%)	9 (16.1%)	0.45
Scheduled for repeat ablation	2 (3.7%)	2 (3.6%)	1.00
Antiarrhythmic drugs at 1 year - Class l - Class II - Class III - Sotalol - Amiodarone - Class IV - Digoxin	25 (46.3%) 5 (9.3%) 13 (24.1%) 6 (11.1%) 3 (5.6%) 3 (5.6%) 4 (7.4%) 2 (3.7%)	32 (57.1%) 2 (3.6%) 19 (33.9%) 10 (17.9%) 10 (17.9%) 0 (0%) 1 (1.8%) 2 (3.6%)	0.26 0.27 0.26 0.32 0.046 0.12 0.20 1.00
CIED implanted post-procedure - Pacemaker - ICD	1 (1.9%) 1 (1.9%) 0 (0%)	0 (0%) 0 (0%) 0 (0%)	0.49 0.49 -
Long-term complications - Persistent PNP - Pulmonary vein stenosis - Atrioesophageal fistula	0 (0%) 0 (0%) 0 (0%) 0 (0%)	0 (0%) 0 (0%) 0 (0%) 0 (0%)	- - - -
Death	1 (1.9%)	0 (0%)	0.49

Values are presented as mean ± standard deviation or as count (%)

*AF* atrial fibrillation, *AFL* atrial flutter, *AT* atrial tachyarrhythmia, *CBA* cryoballoon ablation, *CIED* cardiac implantable electronic devices, *HPSD* high-power short-duration, *ICD* implantable cardioverter defibrillator, *PNP* phrenic nerve paly, *SVT* supraventricular tachycardia, *vHPSD* very high-power short-duration

Durable PVI on PV level was present in 57.9% in the vHPSD/HPSD group versus 73.5% in the CBA group (*P* = 0.24). On patient-level, the number of patients with durable PVI during a repeat procedure was statistically not different between groups (40% [vHPSD/HPSD] vs. 22% [CBA], *P* = 0.58). Most reconnections occurred in the right-sided PVs (60%) in the vHPSD/HPSD group and in the RSPV in the CBA group (62.5%).

As for the long-term safety profiles, no long-term complications were observed in either group. All 3 cases of temporary PNP in the CBA cohort resolved without permanent sequelae. Finally, during the 12-month follow-up period, there was one case of sudden death with unknown cause in the vHPSD/HPSD group.

## Discussion

Point-by-point RFCA guided by 3D mapping has remained one of the most common modalities for the invasive treatment of AF despite the emergence of new energy sources. The goal of PVI is to create contiguous, irreversible, transmural and durable ablation lesion encircling the PVs, while minimizing collateral damage (e.g., esophagus, coronary arteries, and phrenic nerve). The advent of vHPSD has resulted in more effective ablation lesions in shorter time frames [13, 17]. However, vHPSD lesions are shallower and wider than lesions with conventional or HPSD RFCA settings due to more resistive than conductive tissue heating [12, 18]. By combining the speed and safety of vHPSD for the posterior wall and the lesion depth of AI-guided HPSD for the anterior wall (thicker tissue), we may achieve an optimal ablation setting. vHPSD requires a stable catheter position and good catheter-tissue contact, conditions which may be difficult to obtain on the left atrial appendage-PV ridge. We demonstrated that the use of hybrid vHPSD/HPSD for point-by-point PVI resulted in a similar procedural efficiency and efficacy as CBA with a high first-pass isolation rate. Furthermore, radiation exposure was lower in the hybrid vHPSD/HPSD group in comparison to CBA. These results highlight the potential of hybrid vHPSD/HPSD to perform efficient and effective PVI procedures.

HPSD (45–50 W) has improved procedure times and LA dwell times compared with lower power RF settings (25–35 W) with similar or even lower arrhythmia recurrence rates [5, 9]. In comparison to CBA, a meta-analysis showed that HPSD had comparable efficacy and safety profiles as CBA; however, it was associated with longer procedure times [19]. The availability of the QDOT Micro ablation catheter (Biosense Webster), with enhanced temperature monitoring, has made vHPSD (90 W/4 s) possible. The randomized POWER PLUS trial showed that vHPSD resulted in shorter procedure times (70 [60, 80] vs. 75 [65, 88] min) with similar freedom from arrhythmia recurrence in comparison to AI-guided ablation (35–50 W) [13]. However, a recent real-world prospective nonrandomized 2-center study from the UK demonstrated that procedure times with vHPSD were still longer than CBA (110 ± 35 vs 95 ± 20 min, *P* = 0.024) [20]. Interestingly, the FAST AND FURIOUS PVI study demonstrated that mean procedure times with vHPSD can be less than 1 h when the procedure is performed by experienced operators and a “very-close” protocol (3–4 mm ILD at the anterior aspect and 5–6 mm at the posterior aspect of the LA). [11]

It seems that a high first-pass isolation rate is important to achieve short procedure times by eliminating the search for gaps. In the randomized POWER PLUS trial, there as a trend toward lower rates of first-pass isolation in the vHPSD group (84% vs. 90%, *P* = 0.09) probably due to the more shallow ablation lesions with vHPSD [13]. Kariki et al. demonstrated that a hybrid vHPSD/HPSD approach was associated with a higher first-pass isolation rate than vHPSD only (73% vs. 51%, *P* = 0.02) [14]. This approach combines the more shallow and wider ablation lesions of vHPSD on the posterior wall with deeper lesions achieved of HPSD anteriorly.

Our results shows that a hybrid vHPSD/HPSD approach under general anesthesia can result in PVI procedures within 1 h with a high first-pass isolation rate (80%) when performed by experienced operators. Furthermore, procedure times with vHPSD/HPSD showed similar low variability as CBA with an interquartile range of only 16 min for both techniques. Although the PVI durability during repeat procedures was numerically lower than CBA (58% vs. 74%), this was not statistically significant. The benefit of RFCA compared to CBA is that it is associated with a lower radiation exposure, provides additional information by using 3D mapping (e.g., presence of low-voltage areas, PV anatomy), is more versatile (e.g., concomitant treatment of atrial flutter, posterior wall isolation), and prevents certain CBA-specific complications (e.g., PNP, gastric paresis). General anesthesia was preferred in our RFCA procedures to prevent map shifts and to provide more catheter stability using low tidal volumes (3–3.5 ml/kg). The use of general anesthesia in PVI procedures has been associated with better arrhythmia outcomes than conscious sedation [21, 22]. Interestingly, in our study, the use of general anesthesia did not prolong the cathlab time in comparison to CBA procedures with deep sedation.

In the future, PVI with hybrid vHPSD/HPSD approach can even become more personalized. For example, in the QDOT-by-LAWT randomized trial the use of vHPSD was based on LA wall thickness (LAWT) as measured by CT [23]. LAWT-guided PVI combining vHPSD and standard-power ablation was not inferior to the CLOSE protocol in terms of 1-year atrial arrhythmia-free survival and demonstrated a reduction in procedural and RF times.

## Study limitations

The main limitations of the current study is the retrospective, observational, single-center study design which may impact generalization of our conclusions. There was selection bias as demonstrated by the higher incidence of common ostia PV and larger LA dimensions in the hybrid vHPSD/HPSD group. However, despite the more complex LA anatomy, the procedure times in the hybrid vHPSD/HPSD were comparable to the CBA group when performed by experienced operators. There is limited data on the PVI durability therefore strong statements regarding PVI durability of the hybrid vHPSD/HPSD approach are not possible. Finally, the small sample size precludes strong conclusions regarding safety in both groups.

## Conclusion

Point-by-point PVI using radiofrequency energy has evolved continuously in the last decade with the advent of irrigation, wide antral circumferential ablation, contact-force, CLOSE-protocol, and HPSD [13, 24]. The hybrid use of vHPSD/HPSD seems to be an optimal RF ablation technique creating a good balance between procedural efficiency, efficacy and safety. Despite the availability of single-shot techniques, the versality of a focal RF ablation catheter renders its use still popular among experienced operators. We demonstrated that the use of hybrid vHPSD/HPSD ablation strategy was as fast and effective as CBA for achieving PVI. The future will show whether further improvements in RFCA are possible or if we have reached our limits with this technique.

## Abbreviations

AF: Atrial fibrillation
AI: Ablation index
CBA: Cryoballoon ablation
HPSD: High-power short-duration
LA: Left atrium
LAWT: Left atrial wall thickness
PFA: Pulsed field ablation
PNP: Phrenic nerve palsy
PV: Pulmonary vein
PVI: Pulmonary vein isolation
RF: Radiofrequency
RFCA: Radiofrequency catheter ablation
TTI: Time-to-isolation
vHPSD: Very high-power short-duration
WACA: Wide antral circumferential ablation
